# Renal and hepatic function of patients with severe tricuspid regurgitation undergoing inferior caval valve implantation

**DOI:** 10.1038/s41598-021-01322-2

**Published:** 2021-11-08

**Authors:** Bernd Hewing, Isabel Mattig, Fabian Knebel, Verena Stangl, Michael Laule, Karl Stangl, Henryk Dreger

**Affiliations:** 1 Corporate Member of Freie Universität Berlin and Humboldt-Universität Zu Berlin, Medizinische Klinik Mit Schwerpunkt Kardiologie und Angiologie, Charité—Universitätsmedizin Berlin, Campus Charité Mitte, Charitéplatz 1, 10117 Berlin, Germany; 2 Berlin Institute of Health, Charité—Universitätsmedizin Berlin, Berlin, Germany; 3 DZHK (German Centre for Cardiovascular Research), Partner Site, Berlin, Germany; 4 Department of Cardiology III—Adult Congenital and Valvular Heart Disease, University Hospital Muenster, Muenster, Germany

**Keywords:** Cardiology, Interventional cardiology

## Abstract

Due to progressive abdominal-venous congestion severe tricuspid regurgitation (TR) is a common cause of cardiorenal and cardiohepatic syndrome. We initiated the TRICAVAL study to compare interventional valve implantation into the inferior vena cava (CAVI) versus optimal medical therapy (OMT) in severe TR. In the present subanalysis, we aimed to evaluate the effects of CAVI on clinical signs of congestion, renal and hepatic function. TRICAVAL was an investigator-initiated, randomized trial. Twenty-eight patients with severe TR were randomized to OMT or CAVI using an Edwards Sapien XT valve. Probands who completed the 3-month follow-up (CAVI [*n* = 8], OMT [*n* = 10]) were evaluated by medical history, clinical examination, and laboratory testing at baseline, 3 and 12 months. After 3 months, the CAVI group exhibited a significant reduction of body weight (from 80.7 [69.0–87.7] kg to 75.5 [63.8–84.6] kg, *p* < 0.05) and abdominal circumference (from 101.5 ± 13.8 cm to 96.3 ± 15.4 cm, *p* ≤ 0.01) and a trend to lower doses of diuretics compared to OMT. Renal and hepatic function parameters did not change significantly. Within a short-term follow-up, CAVI led to an improvement of clinical signs of venous congestion and a non-significant reduction of diuretic doses compared to OMT.

## Introduction

Severe tricuspid regurgitation (TR) is associated with high morbidity and mortality^1,2^. It exacerbates right heart failure and patients with severe TR typically exhibit an increased demand for diuretic therapy^1^. Right heart and kidney function are closely related with each other and severe TR associated right heart failure may finally lead to renal function impairment^3–6^. The mutually influencing dysfunction of both organs is subsumed under the term cardiorenal syndrome^7^. Analogous, cardiohepatic syndrome describes an association between heart failure and hepatic malfunction^8^. Severity of TR was described as an independent risk factor for hepatic dysfunction, which implies liver deterioration due to abdominal-venous congestion^9^.

Symptomatic medical therapy and surgical reconstruction, or replacement of the tricuspid valve are recommended for the treatment of severe TR according to current guidelines^10^. Novel interventional therapies such as cava valve implantation are being evaluated as alternative treatment options for severe TR^11–14^.

First-in-human studies and a case series investigated the effects of bioprosthetic valve implantation into the inferior vena cava or in both—inferior and superior vena cava^15–18^. These studies suggested an improvement of symptoms of right heart failure^15,16^ and a reduction of venous congestion^15–18^. However, a focused investigation on the effect of vena cava valve implantation on renal and hepatic function is still lacking. On the assumption that a singular implantation of an Edwards Sapien XT valve into the inferior vena cava in patients with severe TR leads to an improvement of abdominal-venous congestion, the TRICAVAL trial (Treatment of Severe Secondary Tricuspid Regurgitation in Patients with Advance Heart Failure with Caval Vein Implantation of the Edwards Sapien XT Valve, NCT02387697) was initiated^19^. Patients were randomized either to valve implantation into the inferior vena cava (CAVI) or optimal medical therapy (OMT)^19^. All valve implantations were performed successfully^20^. Nevertheless, two valve dislocations and two stent migrations occurred within the first 48 h after implantation^20^. Therefore, the study was stopped due to safety concerns^20^. The present subanalysis of TRICAVAL aimed to evaluate the effect of CAVI on clinical signs of congestion as well as on renal and hepatic function parameters.

### Methods

#### Study design

The study design of TRICAVAL was previously published by Dreger et al.^20^. Briefly, TRICAVAL was an investigator-initiated, randomized trial performed in accordance with relevant guidelines and regulations including the Declaration of Helsinki.

**Figure 1:**
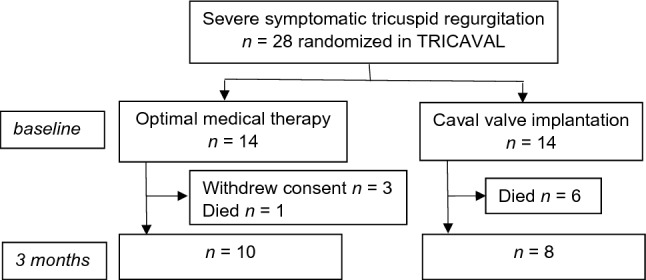
TRICAVAL flow chart from enrolment at baseline to 3-month follow-up.

From 2015 to 2017, 28 patients with severe secondary TR, New York Heart Association (NYHA) class ≥ II, optimal medical treatment and high surgical risk were randomized to CAVI (*n* = 14) or OMT group (*n* = 14) (Fig. 1). Exclusion criteria included regular dialysis or serum creatinine above 3 mg/dl. In the CAVI group, Edwards Sapien XT valves (Edwards Lifesciences, Irvine, CA, USA) were implanted via transfemoral access. As previously reported, 18 patients completed the 3-month follow-up (FUP) after six deaths in the CAVI group and one death and three withdrawals of consent in the OMT group^20^. Twelve patients with six participants in each group were examined 12 months after enrolment^20^. Heart failure medication at baseline was published previously^20^.

At baseline, 3- and 12-month FUP, patients were evaluated by medical history taking and clinical examination including body weight, abdominal and lower leg circumference, blood pressure and heart rate measurements. Body weight was collected from undressed patients using the same scale mostly in the morning. The abdominal circumference was measured slightly above the hip, the leg circumferences 10 cm above the malleolus. The sum of both leg circumferences was used for further analysis. Laboratory testing of venous blood samples comprised creatinine, cystatin C, urea, protein, albumin, alanine aminotransferase (ALT), aspartate aminotransferase (AST), gamma-glutamyl transpeptidase (gamma-GT) and bilirubin. Glomerular filtration rate (GFR) was estimated using creatinine according to the Chronic Kidney Disease Epidemiology Collaboration (CKD-EPI equation)^21^ and using cystatin C calculated by the equation “GFR [ml/min] = 74.835/cystatin C [mg/l]^1.333^”^22^. Albumin was measured in 24 h-urine.

In accordance with the National Kidney Foundation, chronic kidney disease was graded in five stages (CKD stages): kidney damage with normal GFR (≥ 90 ml/min/1.73 m^2^, stage I), mild (60–89 ml/min/1.73 m^2^, stage II), moderate (30–59 ml/min/1.73 m^2^, stage III), and severe GFR reduction (15–29 ml/min/1.73 m^2^, stage IV) as well as kidney failure (< 15 ml/min/1.73 m^2^ or dialysis, stage V)^23^.

### Statistical analyses

Sample size of the TRICAVAL study was calculated taking into account the maximal oxygen uptake after 3 months (primary endpoint). An intergroup difference of 8 ml kg^−1^ min^−1^ was defined as clinically significant^20^. A study cohort of 34 patients was estimated based on a T test, a *P* level < 0.05, a power of 80%, and an assumed standard deviation of 8 ml kg^−1^ min^−1^ (nQuery Advisor 7.0; Statistical Solutions Ltd, Cork, Ireland)^20^. Including a drop-out rate of 15%, 40 patients were designated for randomization^20^. Further statistical analyses were carried out using SPSS Statistics version 25 (IBM Corp, New York, NY, USA). Continuous variables are shown as mean and standard deviation or median and interquartile ranges depending on the distribution of parameters (uniform per variable); categorical variables are given as absolute number with percentages. The distribution of parameters (normal versus not normal) was evaluated by skewness. For intergroup analyses, independent-samples *t* test was used for normally distributed continuous parameters and Mann–Whitney-*U*-test for not normally distributed continuous parameters. To compare values between baseline and 3-month as well as 12-month FUP time-points, paired-samples *t* test was used for normally distributed continuous parameters and Wilcoxon-test for not normally distributed continuous parameters. As the variables were mainly used to generate hypotheses, an adjustment of the *P* values was not carried out. Categorical variables were evaluated by Chi squared test using Fischer’s exact test. *P* < 0.05 was considered statistically significant.

### Ethics approval

The TRICAVAL study was approved by the local ethics committee (Landesamt für Gesundheit und Soziales Berlin, Germany) and state authorities (Bundesinstitut für Arzneimittel und Medizinprodukte, Bonn, Germany).

### Consent to participate/consent for publication

Written informed consent was obtained from each participant.

## Results

Baseline characteristics of the 18 participants of the TRICAVAL study, who completed the 3-month FUP, are shown in Table 1. Left ventricular function and tricuspid annular plane systolic excursion (TAPSE, as a parameter for right ventricular function) were mainly within a normal range and did not differ between both groups. None of the participants reported a prior stroke or peripheral vascular disease in their medical history.

**Table 1:** Baseline characteristics.

	OMT (*n* = 10)	CAVI (*n* = 8)	*P* value
Female sex, n (%)	5 (50)	6 (75)	1.000
Age, years (IQR)	78 (73.3–83.9)	79 (68.3–82.6)	0.965
**NYHA class, n (%)**			1.000
1	0 (0.0)	0 (0.0)	
2	1 (10.0)	1 (12.5)	
3	9 (90.0)	7 (87.5)	
4	0 (0.0)	0 (0.0)	
Logistic EuroSCORE I, % (IQR)	10.1 (8.1–21.0)	15.6 (9.6–29.7)	0.653
BMI, kg/m^2^ (IQR)	25.0 (21.4–27.4)	27.1 (24.9–30.4)	0.091
LVEF, % (IQR)	60.0 (54.3–61.3)	60.0 (52.5–62.0)	0.785
TAPSE, mm (IQR)	15.0 (11.8–22.0)	16.5 (13.3–18.0)	0.721
EROA, cm^2^ (IQR)	0.8 (0.7–1.5)	1.0 (0.5–1.7)	0.929
VC, mm (IQR)	12.0 (8,5–13.25)	13.0 (12.3–19.0)	0.067
TR Vmax, m/s (IQR)	2.65 (1.98–3.45)	2.4 (2.2–2.6)	0.593
NT-proBNP, ng/l (IQR)	2233.0 (1596.3–3954.0)	2342.0 (1404.8–2740.3)	0.657
Coronary artery disease, n (%)	5 (50.0)	3 (37.5)	0.664
Arterial hypertension, n (%)	8 (80.0)	7 (87.5)	1.000
Diabetes mellitus, n (%)	4 (40.0)	3 (37.5)	1.000

Continuous variables are shown as median and interquartile ranges, categorical variables are given as absolute number with percentages.

CAVI, caval valve implantation; OMT, optimal medical therapy; NYHA class, New York Heart Association Class; Logistic EuroSCORE I, Logistic European System for Cardiac Operative Risk Evaluation I; BMI, body mass index; LVEF, left ventricular ejection fraction; TAPSE, tricuspid annular plane systolic excursion; EROA, effective regurgitant orifice area; VC, vena contracta; TR Vmax, maximal tricuspid regurgitation velocity; NT-proBNP, N-terminal pro brain natriuretic peptide.

### Clinical signs of congestion

**Figure 2:**
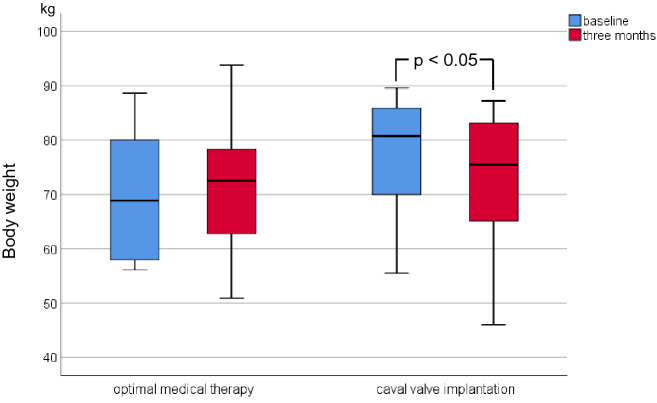
Body weight of patients at baseline and 3-month follow-up.

**Figure 3:**
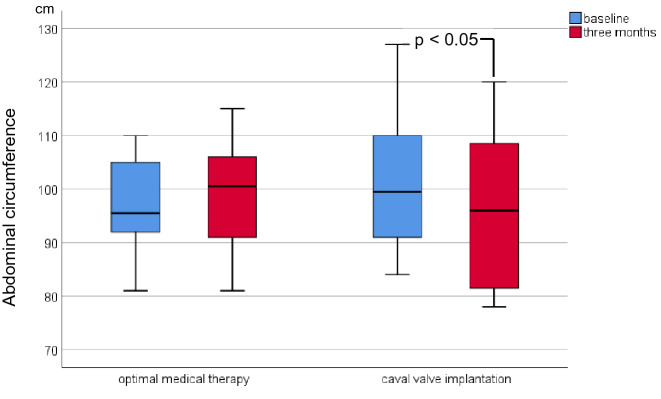
Abdominal circumference of patients at baseline and 3-month follow-up.

**Figure 4:**
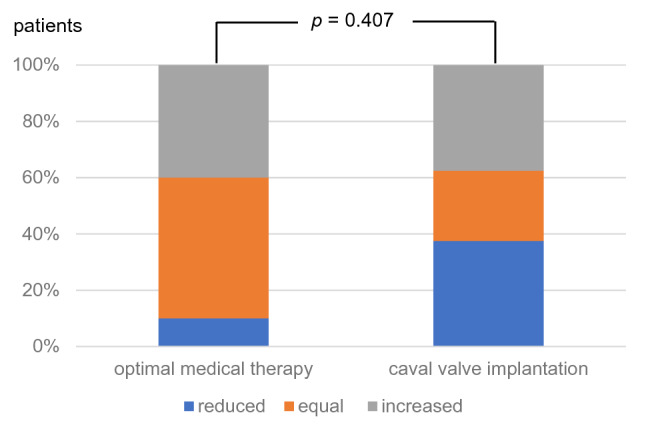
Course of diuretic doses between baseline and 3-month follow-up.

**Figure 5:**
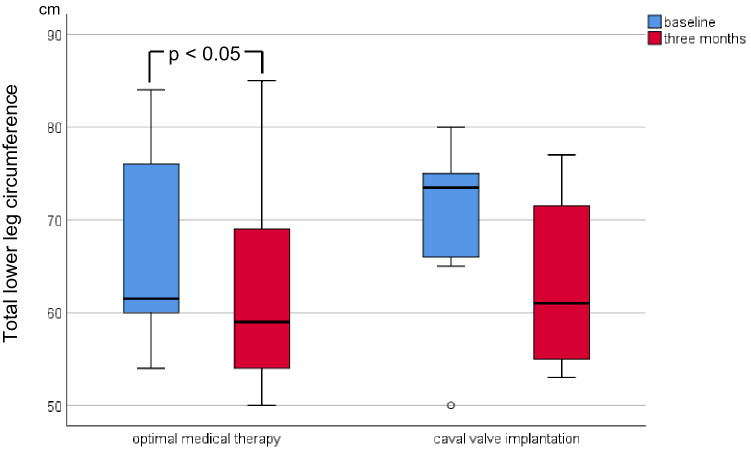
Lower leg circumference (as sum of both leg circumferences) of patients at baseline and 3-month follow-up.

At 3-month FUP, the CAVI group exhibited a significantly lower body weight and reduced abdominal circumference compared to baseline (Table 2; Figs. 2, 3), while in the OMT group both parameters remained unchanged. Furthermore, there was a higher proportion of participants with diuretic dose reduction in the CAVI group compared to the OMT group (Fig. 4). Total lower leg circumference was lower in both groups at 3-month FUP, but only reached statistical significance in the OMT group (Fig. 5). After 12 months, six patients of the CAVI group, who completed 12-month FUP, showed a sustained trend to lower body weight as well as reduced abdominal and leg circumference. Overall, however, no significant intra- or intergroup difference regarding clinical signs of congestion were detected (Table 4).

**Table 2:** Haemodynamic parameters at baseline and 3-month follow-up.

	OMT		CAVI	
	Baseline	3 months	Baseline	3 months
Systolic blood pressure, mmHg ± SD	115.0 ± 8.2	116.0 ± 16.3	115.0 ± 11.3	113.1 ± 10.0
Diastolic blood pressure, mmHg ± SD	72.5 ± 8.9	64.5 ± 6.4*	64.4 ± 9.0	61.9 ± 5.9
Heart rate/min (IQR)	80.0 (70.5–82.5)	73.0 (64.8–85.3)	66.0 (60.5–80.0)	71.0 (67.0–84.3)

Continuous variables are shown as mean and standard deviation (SD) or median and interquartile ranges (IQR) depending on the distribution of parameters (uniform per variable).

OMT, optimal medical therapy; CAVI, caval valve implantation.

*p < 0.05 compared to baseline.

### Renal function

There was no significant change in levels of laboratory parameters including serum creatinine, cystatin C, urea, serum protein, serum albumin as well as calculated GFR (based on creatinine and cystatin C) between baseline and 3-month FUP within each group, nor a significant difference in these parameters between both groups at 3-month FUP (Table 3). 24 h-albumin urine was available in 5 patients in the OMT group and 4 patients in the CAVI group and did not change significantly between baseline and 3-month FUP (*p* = 0.225 for OMT group, *p* = 0.144 for CAVI group).

**Table 3:** Renal and hepatic parameters at baseline and 3-month follow-up.

	OMT (*n* = 10)		CAVI (*n* = 8)	
	Baseline	3 months	Baseline	3 months
Serum creatinine, mg/dl ± SD	1.4 ± 0.4	1.7 ± 0.7	1.6 ± 0.6	1.5 ± 0.5
GFR (creatinine), ml/min (IQR)	46.5 (30.0–56.0)	36.5 (23.5–58.8)	36.5 (24.5–62.8)	35.5 (28.0–60.8)
Urea, mg/dl (IQR)	73.5 (47.8–150.8)	83.5 (55.5–143.8)	81.5 (40.8–144.8)	63.5 (46.8–124.8)
Cystatin C, mg/l ± SD	1.9 ± 0.6	2.1 ± 0.7	2.1 ± 1.0 (n = 7)	2.1 ± 0.8 (n = 7)
GFR (cystatin C), ml/min ± SD	34.9 ± 13.1	32.6 ± 14.1	36.8 ± 20.9 (n = 7)	34.1 ± 16.0 (n = 7)
Serum protein, g/l (IQR)	75.5 (66.3–79.3)	75.5 (68.0–79.8)	67.5 (65.5–69.8)	69.5 (67.3–79.5)
Serum albumin, g/l ± SD	41.2 ± 5.1	42.0 ± 3.2	39.7 ± 3.2	39.5 ± 5.3
ALT, U/l (IQR)	17.0 (13.8–29.3)	16.0 (14.8–24.0)	26.0 (11.8–30.8)	18.0 (14.3–23.8)
AST, U/l ± SD	29.2 ± 8.6	31.4 ± 9.3	29.3 ± 8.2	28.4 ± 5.4
Gamma-GT, U/l (IQR)	226.0 (101.5–264.0) (n = 9)	144.0 (70.5–217.0) (n = 9)*	87.5 (52.3–155.3)	103.5 (62.3–193.0)
Bilirubin, mg/l (IQR)	0.9 (0.7–1.1)	0.9 (0.5–1.3)	0.7 (0.4–0.9)	0.5 (0.3–1.0)

Continuous variables are shown as mean and standard deviation (SD) or median and interquartile ranges (IQR) depending on the distribution of parameters (uniform per variable).

OMT, optimal medical therapy; CAVI, caval valve implantation; GFR, glomerular filtration rate; ALT, alanine aminotransferase; AST, aspartate aminotransferase; Gamma-GT, gamma-glutamyl transpeptidase; n, number of examined patients with completed 3-month follow-up.

*p < 0.05 compared to baseline.

After 12 months, the OMT group exhibited a significant increase in cystatin C associated with a decrease in calculated GFR and higher levels of serum albumin compared to baseline. No further significant intra- or intergroup differences were observed at the 12-month FUP (Table 4).

**Table 4:** Clinical signs of venous congestion, renal and hepatic parameters at baseline and 12-month follow-up.

	OMT (*n* = 6)		CAVI (*n* = 6)	
	Baseline	12 months	Baseline	12 months
Body weight, kg ± SD	68.4 ± 12.8	70.7 ± 13.0	74.6 ± 12.1	72.6 ± 16.3
Abdominal circumference, cm ± SD	97.3 ± 10.3	98.0 ± 11.6	97.3 ± 10.8	96.2 ± 13.2
Total lower leg circumference, cm (IQR)	60.5 (54.8–66.6)	56.5 (49.0–66.8)	73.5 (61.3–76.3)	61.5 (48.5–76.5)
Serum creatinine, mg/dl ± SD	1.2 (1.0–1.6)	1.2 (1.0–1.7)	1.6 (1.1–1.9)	1.5 (1.1–2.4)
GFR (creatinine), ml/min (IQR)	50.0 ± 16.6	47.5 ± 15.4	39.7 ± 17.3	38.2 ± 16.9
Urea, mg/dl (IQR)	64.5 (39.3–99.3)	82.5 (47.8–101.3)	81.5 (32.8–115.0)	70.0 (41.3–100.8)
Cystatin C, mg/l ± SD	1.4 (1.3–2.2) (*n* = 5)	1.5 (1.4–2.3)* (*n* = 5)	2.2 (1.2–3.2)	2.1 (1.4–3.2)
GFR (cystatin C), ml/min ± SD	47.0 (24.5–56.2)	42.9 (21.7–47.1)*	24.2 (15.0–54.8)	25.8 (15.0–49.2)
Serum protein, g/l (IQR)	73.0 (62.5–79.0) (*n* = 5)	73.0 (63.5–83.0) (*n* = 5)	68.5 (66.5–72.0)	72.0 (69.0–76.3)
Serum albumin, g/l ± SD	42.0 (35.0–43.0) (*n* = 5)	46.2 (38.6–49.9)* (*n* = 5)	40.4 (36.3–43.2)	39.6 (35.9–42.1)
ALT, U/l (IQR)	17.0 (13.8–34.3)	20.5 (17.0–27.8)	29.0 (15.0–31.3)	16.5 (11.5–22.8)
AST, U/l ± SD	29.5 ± 9.6	31.7 ± 8.0	30.0 ± 6.6	27.7 ± 7.7
Gamma-GT, U/l (IQR)	226.0 (86.0–872.0) (*n* = 5)	166.0 (64.5–248.0) (*n* = 5)	64.0 (51.0–116.0)	65.0 (55.3–105.5)
Bilirubin, mg/l (IQR)	0.8 (0.7–1.0)	0.8 (0.6–1.3)	0.7 (0.4–0.9)	0.5 (0.4–0.7)

Continuous variables are shown as mean and standard deviation (SD) or median and interquartile ranges (IQR) depending on the distribution of parameters (uniform per variable).

OMT, optimal medical therapy; CAVI, caval valve implantation; GFR, glomerular filtration rate; ALT, alanine aminotransferase; AST, aspartate aminotransferase; Gamma-GT, gamma-glutamyl transpeptidase; n, number of examined patients with completed 12-month follow-up.

*p < 0.05 compared to baseline.

**Figure 6:**
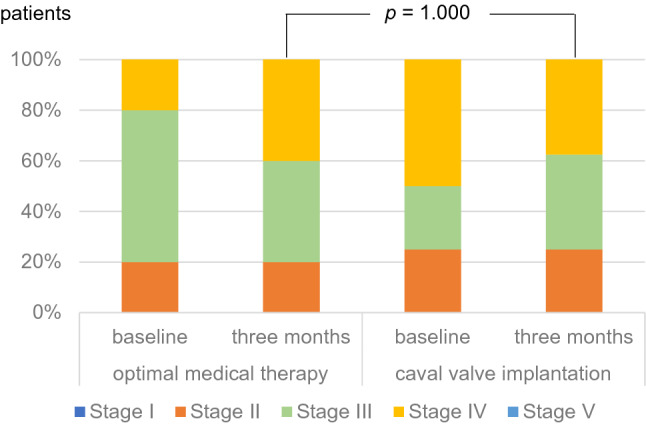
Distribution of chronic kidney disease stages (in accordance with the National Kidney Foundation) in patients of the optimal medical therapy (*n* = 10) or caval valve implantation (*n* = 8) group at baseline and 3-month follow-up.

At baseline, stages of renal failure showed the following distribution: 25% stage II, 37.5% stage III, and 37.5% stage IV in the CAVI group and 20% stage II, 60% stage III, and 20% stage IV in the OMT group. After 3 months, one patient in the CAVI group improved from CKD stage from IV to III, while two patients in the OMT group showed a deterioration from CKD stage III to IV (Fig. 6).

### Hepatic function

In the CAVI group, liver function as measured by ALT, AST, gamma-GT and bilirubin remained stable after 3 and 12 months compared to baseline. Furthermore, there was no significant difference in these parameters between both groups at the 3- and 12-month FUP (Tables 3, 4).

### Discussion

This subanalysis of the TRICAVAL study represents the first controlled, prospective, randomized study to evaluate renal and hepatic function in patients undergoing CAVI compared to an OMT control group. We found that renal and hepatic function remained unchanged in both groups at 3-month FUP, but deteriorated further over time in the OMT control group. The CAVI group exhibited a significantly lower body weight paralleled by a reduction of diuretic doses after 3 months, both suggesting a decrease of TR-induced venous congestion. These results extend our previously published echocardiographic data of TRICAVAL, which demonstrated decreased systolic hepatic vein reflux volume and hepatic vein diameter after CAVI^24^. However, TR severity as well as right heart morphology and function showed no significant differences in the intergroup comparison at the 3-month follow-up^24^. After CAVI, vena contracta (3-month FUP: 15.2 [9.1–21.0] mm) and effective regurgitant orifice area did not change significantly^24^. Similarly, TAPSE (3-month FUP: 16.0 [13.0–18.8] mm) remained unchanged^24^. The reduction of abdominal-venous congestion as shown in both clinical and echocardiographic data implies sufficient sealing of the inferior vena cava after valve implantation and thereby the effectiveness of the valve implantation. This was additionally reflected by improved symptoms of abdominal-venous congestion and increased quality of life after CAVI at 3-month FUP^19,20^.

Based on the reduction of TR-induced congestion after CAVI, we hypothesized that renal function would improve—particularly since a pathophysiological connection between congestion and deterioration of renal function is well established^3,4,25–28^. The interaction between haemodynamic changes and renal function was reported by Firth et al. using an isolated rat kidney^27^. A stepwise increase in venous pressure in the presence of stable arterial perfusion resulted in a decrease in GFR, sodium excretion and fractional sodium excretion^27^. The worsening of renal function was reversible after lowering venous pressure^27^. The development of renal dysfunction caused by haemodynamic changes in the venous system was also detected in patients with TR and with decompensated heart failure^26,28^. In advanced cardiac decompensation due to heart failure with reduced ejection fraction, an elevated central venous pressure measured with a balloon-tipped catheter predicted a deterioration in renal function^28^. Measurements were performed at baseline and discharge after intensive care treatment with diuretics and intravenous vasodilators and, at both time-points, an elevated central venous pressure but not a reduced cardiac index was associated with renal worsening^28^. In the present study, the reduction in diuretic doses after CAVI may indicate a functional improvement of fluid balance by the kidneys. Creatinine and cystatin C levels as well as calculated GFR, remained unchanged 3 and 12 months after CAVI, while calculated GFR using cystatin C decreased at 12 months in the OMT group.

In accordance with our findings, other interventional TR studies did not describe an improvement of renal function after TR therapy: a recent study with single and bicaval valve implantation in patients with severe symptomatic TR reported a postprocedural deterioration of renal function (increase in creatinine levels), which may partly be explained by the application of contrast medium during the intervention^18^. Analogous to our findings, a postprocedural reduction of abdominal congestion was observed^18^. Studies investigating a different interventional therapeutic approach of severe TR treatment—edge-to-edge therapy by implantation of MitraClips—demonstrated a stable renal function (creatinine, GFR, urea) and constant diuretic doses prior to discharge and 6 months after intervention^13,29^. Prior to discharge following tricuspid edge-to-edge therapy, the diameter of the inferior vena cava remained unchanged after intervention versus baseline indicating a smaller reduction in abdominal-venous congestion compared to CAVI^13^; this discrepancy may be an explanation for the different findings on diuretic doses between both interventional approaches.

Our subanalysis thus demonstrates an effective reduction of abdominal-venous congestion after heterotopic valve implantation. Improvements in the implantation technique using dedicated devices like TricValve (P&F, Vienna, Austria) have proven to be technically safe^17^. Heart failure patients frequently present in advanced stages of TR with large defects not amenable for interventional repair by edge-to-edge techniques or annuloplasty. Therefore, improving symptoms and outcome of these patients remains an unmet clinical need. While our approach was associated with major complications, CAVI as a principle might confer some benefit on patients in advanced stages of TR with significant systolic hepatic vein reflux, who are anatomically unsuitable for other interventional therapies.

In the present study, hepatic parameters remained stable after CAVI. Severity of TR is associated with laboratory changes in gamma-GT and bilirubin due to congestive cardiac hepatopathy^9^. A first study on tricuspid edge-to-edge valve repair reported an improvement of AST, ALT and bilirubin in patients with abnormal hepatic function 6 months after intervention^29^. These results imply a potentially reversible hepatopathy after interventional TR therapy. In the CAVI group of the present study, hepatic parameters were mainly within a normal range at baseline indicating no advanced hepatopathy in our cohort. Accordingly, significant improvements within the normal range may not be expected.

There are some limitations to the present study. Following major complications, including two valve dislocations and two stent migrations in the CAVI group, the study was stopped prematurely resulting in a small study sample size for the present subanalysis. Therefore, our findings can only be considered hypothesis-generating and require confirmation by further studies using dedicated devices with improved safety. Due to the short FUP period and only two-time laboratory FUP-measurement, potential changes in laboratory parameters may have been missed. Short-term fluctuations as well as long-term effects were not considered in the subanalysis. The decrease in body weight and abdominal circumference may be influenced by cardiac cachexia, which occurs particularly in advanced stages of heart failure. As baseline laboratory parameters of hepatic function were mostly within the normal range, no conclusions can be drawn for TR patients with severely impaired hepatic function.

## Conclusion

CAVI leads to a reduction of clinical signs of congestion and reduction of diuretic doses in patients with severe TR. Over the next few years, novel catheter-based techniques will provide more information on long-term effects of interventional TR treatment on renal and hepatic function.
